# Evaluation of *PRNP* Expression Based on Genotypes and Alleles of Two Indel Loci in the Medulla Oblongata of Japanese Black and Japanese Brown Cattle

**DOI:** 10.1371/journal.pone.0018787

**Published:** 2011-05-18

**Authors:** George Msalya, Takeshi Shimogiri, Shotaro Ohno, Shin Okamoto, Kotaro Kawabe, Mitsuru Minezawa, Yoshizane Maeda

**Affiliations:** 1 United Graduate School of Agriculture, Kagoshima University, Kagoshima City, Japan; 2 Faculty of Agriculture, Kagoshima University, Kagoshima City, Japan; 3 Frontier Science Research Center, Kagoshima University, Kagoshima City, Japan; 4 National Institute of Agrobiological Sciences, Tsukuba, Japan; 5 Heifer Breeding Project, NARCO Kikulula-Karagwe, Kagera, Tanzania; Public Library of Science, United Kingdom

## Abstract

**Background:**

Prion protein (PrP) level plays the central role in bovine spongiform encephalopathy (BSE) susceptibility. Increasing the level of PrP decreases incubation period for this disease. Therefore, studying the expression of the cellular PrP or at least the messenger RNA might be used in selection for preventing the propagation of BSE and other prion diseases. Two insertion/deletion (indel) variations have been tentatively associated with susceptibility/resistance of cattle to classical BSE.

**Methodology/Principal Findings:**

We studied the expression of each genotype at the two indel sites in Japanese Black (JB) and Japanese Brown (JBr) cattle breeds by a standard curve method of real-time PCR. Five diplotypes subdivided into two categories were selected from each breed. The two cattle breeds were considered differently. Expression of *PRNP* was significantly (*p*<0.0001) greater in the homozygous deletion genotype at the 23-bp locus in JB breed. Compared to the homozygous genotypes, the expression of *PRNP* was significantly greater in the heterozygous genotype at the 12-bp locus in JB (*p*<0.0001) and in JBr (*p* = 0.0394) breeds. In addition, there was a statistical significance in the *PRNP* levels between the insertion and the deletion alleles of the 23-bp locus in JB (*p* = 0.0003) as well as in JBr (*p* = 0.0032). There was no significance in relation to sex, age, geographical location or due to their interactions (*p*>0.05).

**Conclusion:**

Our results suggest that the del/del genotype or at least its del allele may modulate the expression of *PRNP* at the 23-bp locus in the medulla oblongata of these cattle breeds.

## Introduction

Genetic variations in the prion protein gene (*PRNP*) are linked to the occurrence of transmissible spongiform encephalopathies (TSEs) also called prion diseases in humans, sheep and mice [1]–[4]. TSEs are characterized by abnormal deposits of a protease-resistant isoform of the host genome encoded prion protein (PrP) and are unique in that they can manifest through acquired, inherited, or sporadic origins [5]. Polymorphisms in two regulatory regions in the bovine *PRNP* have been tentatively associated with classical BSE (cBSE) incidences in a few cattle populations [6]–[8]. A 23-bp insertion/deletion (indel) polymorphism in the promoter contains a binding site for the repressor protein 58 (RP58) and a 12-bp indel in intron 1 has a binding site for the transcription factor specificity protein 1 (SP1). The presence or absence of these binding sites modulate the expression of *PRNP* and possibly the expression of PrP in species. Expression of the cellular PrP is necessary for the transmission and propagation of prion diseases [9]. Based on reporter gene assays, increased level of PrP decreases the incubation period for cBSE [10].

BSE cases in Japan rose to 36 in 2010. Of these, 33 cases were reported in Holstein-Friesian (HF) animals while three were reported in local Japanese Black (JB) cattle. So far a number of Japanese cattle populations have been analyzed for various polymorphisms in the *PRNP*. Populations analyzed include the JB breed [11], Japanese Brown (JBr) breed and other local breeds [12], [13]. The aim of the present study was to examine the effect of genotypes of the 23-bp and 12-bp indel loci in the expression of *PRNP* in JB and JBr breeds.

## Results

### DNA genotyping and diplotype analysis

A total of 218 animals including 120 JB and 98 JBr were genotyped for polymorphisms in the 23-bp and 12-bp indel loci of the *PRNP*. Both 23-bp and 12-bp loci were polymorphic in both breeds. At the 23-bp locus, frequency of the deletion homozygous (del/del) was 0.62, that of the heterozygous (ins/del) was 0.30 and that of the insertion homozygous (ins/ins) was 0.08 in JB breed and 0.31, 0.50 and 0.19 respectively in the JBr breed. At the 12-bp locus, the frequencies were 0.39 for the del/del (− −), 0.38 for the ins/del (+−) and 0.23 for the ins/ins (++) in JB breed whereas the frequencies were 0.30, 0.47 and 0.23 respectively in the JBr breed. Compared to the insertion (+) allele, the deletion (−) allele was higher in frequency at both loci in both breeds. JB breed had greater frequencies of the − allele at both loci as compared to the JBr breed. Genotypic and allelic data are presented in Table 1.

**Table 1:** Genotype and gene frequencies of *PRNP* polymorphisms in JB and JBr breeds.

*PRNP* site	Breed	n	genotype frequency			allele frequency	
			+/+	+/−	−/−	+	−
Promoter 23-bp indel	JB	120	0.08	0.30	0.62	0.23	0.77
	JBr	98	0.19	0.50	0.31	0.44	0.56
Intron 12-bp indel	JB	120	0.23	0.38	0.39	0.42	0.58
	JBr	98	0.23	0.47	0.30	0.47	0.53

n: sample size; JB: Japanese Black cattle; JBr: Japanese Brown cattle.

These genotypes were assembled into diplotypes. The left side of these diplotypes represents one of the three genotypes of the 23-bp indel loci whereas the right side constitutes genotypes of the 12-bp indel loci. As shown in Table 2, eight diplotypes were observed in the JB breed whereas seven diplotypes were observed in the JBr breed. The double deletion homozygous (−−/−−) was the highest diplotype (33.33%) in the JB breed whereas the double heterozygous (+−/+−) was the highest diplotype (38.78%) in the JBr. The double insertion homozygous (++/++) diplotype was observed in 16.33% of the JBr animals and in 5.00% of the JB animals. The +−/++ diplotype was observed in 10.00% of the JB animals and 7.14% of the JBr animals while the −−/+− was observed in 20.00% of the JB animals and 5.10% of the JBr animals. The −−/++ diplotype was observed in the JB breed only. Diplotypes ++/+−, ++/−−, +−/−− were rare in both breeds. Considering the breeds together, the −−/−− was the greatest diplotype (29.82%). Five diplotypes namely ++/++, +−/++, −−/++, −−/+−, −−/−− were selected for gene expression analyses in the JB breed and five others (++/+−, +−/+−, −−/+−, +−/++, +−/−−) were selected for the same purpose in the JBr breed. Gene expression was evaluated among these diplotypes to achieve the study objectives.

**Table 2:** Diplotype frequencies for two *PRNP* indel polymorphisms in JB and JBr breeds.

Diplotype	JB	JBr	Total
23 bp/12 bp	n = 120 (%)	n = 98 (%)	n = 218 (%)
++/++	6 ( 5.00)	16 (16.33)	22 (10.09)
++/+−	0 ( 0.00)	3 ( 3.06)	3 ( 1.38)
++/−−	4 ( 3.33)	0 ( 0.00)	4 ( 1.83)
+−/++	12 (10.00)	7 ( 7.14)	19 ( 8.72)
+−/+ −	21 (17.50)	38 (38.78)	59 (27.06)
+−/−−	3 ( 2.50)	4 ( 4.08)	7 ( 3.21)
−−/+ +	10 ( 8.33)	0 ( 0.00)	10 ( 4.59)
− −/+ −	24 (20.00)	5 ( 5.10)	29 (13.30)
− −/− −	40 (33.33)	25 (25.51)	65 (29.82)

n: sample size; (%): percentage of a diplotype; JB: Japanese Black cattle; JBr: Japanese Brown cattle.

### *PRNP* expression based on genotypes of the 23-bp promoter indel

**Figure 1:**
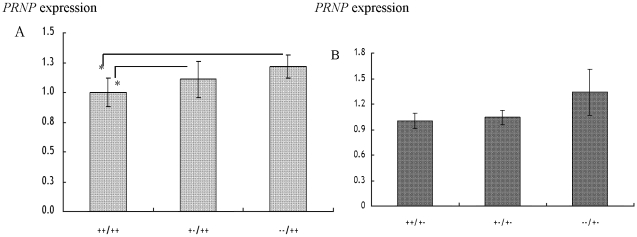
Expression of *PRNP* (mean±SE) in genotypes of the 23-bp promoter indel in A: JB and B: JBr breeds. ++/++ (n = 6); +−/++ (n = 8); −−/++ (n = 8); ++/+− (n = 3); +−/+− (n = 6); −−/+− (n = 5); *significance difference in the *PRNP* expression (*p*<0.05).

To evaluate the expression of *PRNP* among genotypes of the 23-bp promoter indel, three diplotypes were selected from each breed. Diplotypes ++/++ (n = 6), +−/++ (n = 8), and −−/++ (n = 8) were selected in the JB breed whereas diplotypes ++/+− (n = 3), +−/+− (n = 6) and −−/+− (n = 5) were selected in the JBr breed. The left side of these diplotypes constitute of genotypes ++, +− and −− of the 23-bp locus whereas the right which represent the 12-bp locus has a similar genotypes in all diplotypes. It was presumed that a similar genotype at the 12-bp locus would have similar effect in these diplotypes and therefore the gene expression was compared among genotypes of the 23-bp locus on the left side. The 12-bp locus was represented by the ++ genotype in the JB breed and +− in the JBr breed. The expression of *PRNP* relative to the reference *ACTB* gene were analysed. As presented in Table 3, the expression of *PRNP* (mean±SE) were 0.2721±0.033 in the ++/++ diplotype, 0.3017±0.042 in the +−/++ diplotype and 0.3314±0.027 in the −−/++ diplotype in the JB breed. Therefore, the level of *PRNP* was higher in the −−/++ diplotype compared to ++/++ and +−/++ diplotypes suggesting the order of expression of *PRNP* in medulla oblongata of the JB breed as −−/++>+−/++>++/++. The levels of *PRNP* were 0.0801±0.007 in the ++/+− diplotype, 0.0838±0.007 in the +−/+− diplotype and 0.1076±0.022 in the −−/+− diplotype in the JBr breed. The *PRNP* expression was therefore greater in the −−/+− diplotype as compared to the other two diplotypes. The order of expression was −−/+−>+−/+−>++/+− in the JBr breed. Clearly, it can be shown that, diplotypes with the −− genotype had greater *PRNP* levels than those with the ++ or the +− at the 23-bp locus in both breeds (Figure 1). The expression of *PRNP* was significantly different (*p* = 0.0001) among genotypes of the 23-bp locus in JB breed but there was no statistical significance (*p*>0.05) among these genotypes in the JBr breed. Also, there was no significance in relation to sex, age, regions or farms of the animals and their interactions (*p*>0.05).

**Table 3:** *PRNP* expression for various diplotypes in JB and JBr breeds.

Breed	Diplotype	n	Mean *PRNP*	SD	SE
JB	++/++	6	0.2721	0.067	0.033
	+−/++	8	0.3017	0.119	0.042
	−−/++	8	0.3314	0.076	0.027
	−−/+−	10	0.3971	0.134	0.043
	−−/−−	10	0.2552	0.034	0.011
	+−/+−	20	0.0492	0.013	0.003
	−−/+−	20	0.0899	0.039	0.009
JBr	++/+−	3	0.0801	0.012	0.007
	+−/+−	6	0.0838	0.016	0.007
	−−/+−	5	0.1076	0.049	0.022
	+−/++	6	0.1212	0.010	0.004
	+−/+−	12	0.1301	0.052	0.010
	+−/−−	4	0.0984	0.019	0.009
	++/++	8	0.1419	0.018	0.006
	+−/++	7	0.1787	0.040	0.015

n: sample size; *PRNP*: *PRNP*/*ACTB* ratio; SD: Standard deviation; SE: Standard error; JB: Japanese Black cattle; JBr: Japanese Brown cattle.

### *PRNP* expression based on genotypes of the 12-bp indel in intron 1

**Figure 2:**
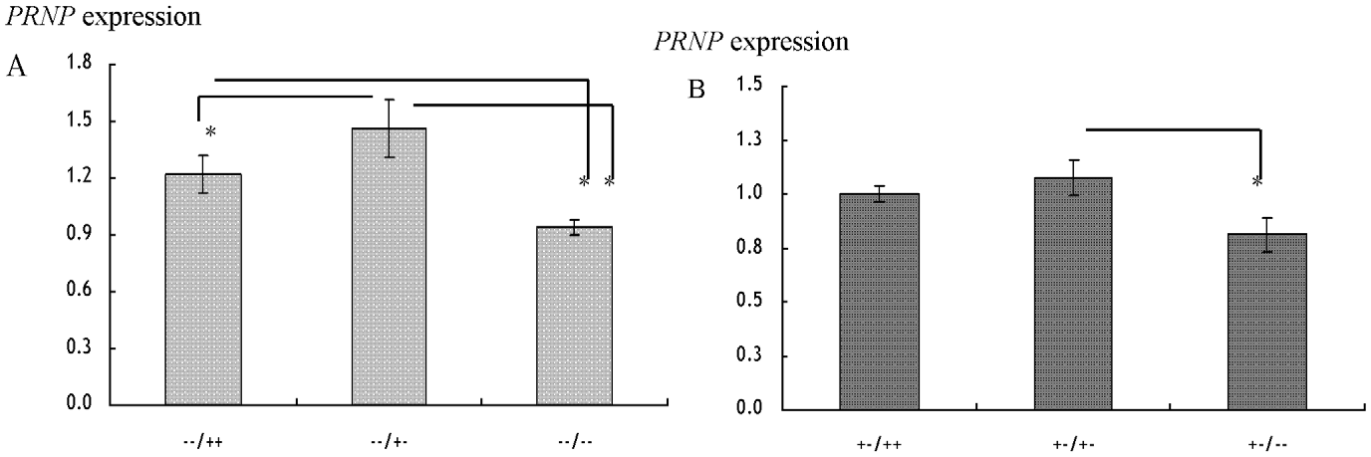
Expression of *PRNP* (mean±SE) in genotypes of the 12-bp intron 1 indel in A: JB and B: JBr breeds. −−/++ (n = 8); −−/+− (n = 10); −−/−− (n = 10); +−/++ (n = 6); +−/+− (n = 12); +−/−− (n = 4); *significance difference in the *PRNP* expression (*p*<0.05).

In order to evaluate *PRNP* expression among the genotypes of the 12-bp locus, we selected animals with diplotypes −−/++ (n = 8), −−/+− (n = 10) and −−/−− (10) from the JB breed. From the JBr breed, animals with diplotypes +−/++ (n = 6), +−/+− (n = 12) and +−/−− (n = 4) were selected. The 23-bp locus was represented by genotypes −− (JB breed) and +− (JBr breed) at the left side of these diplotypes. As summarized in Table 3, the *PRNP* levels were 0.3314±0.027 in the −−/++, 0.3971±0.043 in the −−/+− and 0.2552±0.011 in the −−/−− in the JB breed as well as 0.1212±0.004 in the +−/++, 0.1301±0.010 in the +−/+− and 0.0984±0.009 in the +−/−− in the JBr breed. As a result, animals with the −−/++ diplotype showed the greatest *PRNP* expression followed by those with the −−/+− and −−/−− in the JB breed whereas animals with the +−/++ diplotype showed the greatest *PRNP* expression than those with diplotypes +−/+− and +−/−− in the JBr breed. Our results indicate that, diplotypes with the +− genotype had greater *PRNP* levels compared to those with either ++ or −− at the 12-bp locus in both breeds (Figure 2). The *PRNP* levels were statistically significant among these diplotypes in both JB (*p* = 0.0001) and JBr (*p* = 0.0394) breeds. There was no significant difference in relation to sex, age, regions or farms of the animals and their interactions (*p*>0.05).

### *PRNP* expression with respect to alleles + and − of the 23-bp promoter indel

**Figure 3:**
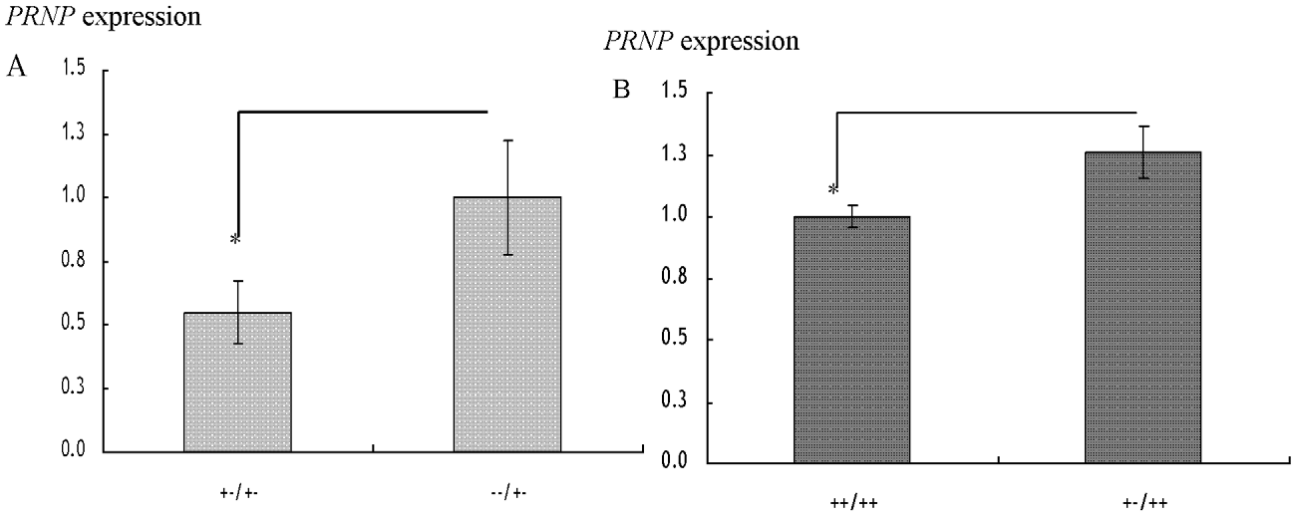
Expression of *PRNP* (mean±SE) with respect to alleles + and − of the 23-bp indel in A: JB and B: JBr breeds. +−/+− (n = 20); −−/+− (n = 20); ++/++ (n = 8); +−/++ (n = 7); *significance difference in the *PRNP* expression (*P*<0.05).

Expression of *PRNP* with respect to alleles + and − of the 23-bp locus were assayed using diplotypes +−/+− and −−/+− in the JB breed and diplotypes ++/++ and +−/++ in the JBr breed. Three of the four alleles in the diplotypes selected in the JB breed were the same (−/+−) so that it was possible to compare the expression of *PRNP* between two alleles. In the JBr breed, except for the alleles of interest the other three alleles were similar (+/++) in both diplotypes thus possible to detect the expression of *PRNP* between alleles + and −. The *PRNP* levels were 0.0492±0.003 and 0.0899±0.009 for the diplotypes +−/+− and −−/+− in the JB breed respectively and 0.1419±0.006 and 0.1787±0.015 respectively for the diplotypes ++/++ and +−/++ in the JBr breed (Table 3). For both breeds, the + allele had a lower expression than those with the − allele (Figure 3). The expression was significant between the two diplotypes in the JB breed (*p* = 0.0003) as well as in the JBr breed (*p* = 0.0032).

## Discussion

Two *PRNP* loci have been tentatively shown to influence the cBSE incidence in some cattle populations [6], [7], [10], [14], [15]–[17]. The −− genotype at the 23-bp and the 12-bp loci were reported to be overrepresented in cBSE-affected cattle [6], [7], [10]. The 23-12- haplotype and a few haplotype tagging single nucleotide polymorphisms (htSNPs) may influence association of the genotype with disease incidence in cattle [15], [16]. Specifically, two htSNPs were shown to influence the association of the 23-bp and 12-bp polymorphisms with the cBSE incidence [16]. Also a −−/−− diplotype has been reported to be significantly overrepresented in BSE-affected animals [7].

We genotyped the 23-bp and 12-bp indel loci in healthy JB and JBr breeds and analysed the expression of its messenger RNA using ten genotype combinations (five diplotypes in JB and five in JBr). The genotypic and allelic data at these loci supplement the data available in Japan for these and other breeds [11], [13], [18]. These data were used in the *PRNP* expression assays and have not been submitted for publication. Our genotypic and allelic frequencies data agree with those published in earlier reports. Notably, the allelic frequencies at the 23-bp locus for the JBr breed resembled to those of the United States (US) HF [19] and to those of the healthy Germany cattle [6]. Allelic frequencies at the 12-bp locus in our breeds were in the same range as those reported for US sires [14], US HF [19] and the United Kingdom (UK) HF [7].

We used the diplotypes to study the effect of the genotypes of the 23-bp and 12-bp loci in the *PRNP* expression. Firstly, we have shown that diplotypes with the −− genotype had higher expression than those with the +− and ++ genotypes at the 23-bp locus in both breeds. Secondly, we showed that diplotypes with the +− genotype had a greater expression than those with the ++ and −− genotypes at the 12-bp locus in both breeds. Considering the genotypes alone, our results suggest the order of *PRNP* expression as −−>+−>++ at the 23-bp locus and +−>++>−− at the 12-bp locus in the medulla oblongata of these breeds. Thirdly, we studied the difference of *PRNP* expression between diplotypes differing in one allele (the + and the −) at the 23-bp locus and showed that diplotypes with the + allele had lower *PRNP* levels compared to those with the − allele.

In summary, the −− genotype was correlated with an increased *PRNP* expression compared to ++ and +− genotypes at the 23-bp locus. This genotype was reported to be significantly overrepresented in BSE-affected cattle presumably due to the homozygous deletion of a *PRNP* transcriptional RP58 binding site [6], [7], [10], [20] at this locus. Surprisingly, the −− genotype at the 12-bp locus which has also been reported to be overrepresented in BSE-affected animals than healthy animals previously [6], [7], [10] showed a lower *PRNP* level compared to genotypes +− and ++. The +− genotype could show high *PRNP* than the other two genotypes due to a possible over-dominance whereby the − allele might be masked by the + allele [21] or other factors including polar over-dominance as reported in other species [22]. There is limited or no information on over-dominance of the + allele at the 12-bp locus in the cattle *PRNP* but compared to the other two genotypes, the +− genotype has been reported to be high in frequency at the 12-bp locus in many cattle populations [17]–[19]. Taking the two loci together, our results thus suggest the −− genotype of the 23-bp locus might contribute a greater *PRNP* level compared to the −− of the 12-bp locus in animals with the −−/−− diplotype as we previously reported [23]. This might be due to the fact that these loci are not entirely independent as they are closely positioned in the region of high LD of the bovine *PRNP* [15]. Thus if proportional to the expression of PrP, the levels of *PRNP* may elucidate the levels of PrP in the medulla oblongata of these animals. We further showed that a different between the + and − alleles at the same position in two diplotypes might significantly affect the level of *PRNP*. Our results showed that an increase of the − allele increased the level of *PRNP* in one of the two diplotypes examined for expression in alleles. If *PRNP* expression is an important factor for the PrP expression, animals with genotypes +− and −− at the 23-bp locus may show higher levels of PrP compared to those with the ++ genotype at this locus. It is worth to note that, the ++/++ diplotype had a low *PRNP* level compared to the other diplotypes in the JB breed an indication that the ++ of both loci may show a low *PRNP* expression. The + variants of either regulatory element have the potential to lower host PrP expression levels [10], thus providing a biological basis for BSE resistance in cattle homozygous for the + [7], [24], [25]. Our expression results agree with those reported for other tissues in other populations *in vivo* and also in the *in vitro* [10].

We report here that the 23-bp − allele and the −− genotype may contribute to a greater *PRNP* expression in the 23/12 diplotypes based assays in the medulla oblongata of JB and JBr breeds. However, the deletion alleles are not entirely independent of one another as there is high linkage disequilibrium (LD) between the two polymorphic sites in *Bos taurus* cattle populations [15], [20]. This suggests that the possible effects of variations in the *PRNP* on incidence of cBSE may be better understood if *PRNP* haplotypes were considered in testing for association with disease incidence. Moreover, *PRNP* haplotypes, containing one or both of the two indel loci, may have a stronger association with either susceptibility or resistance to cBSE than if the indels are considering independently. Also, other functional variants may affect the expression of *PRNP* and therefore carrying out confirmatory studies in these loci or other polymorphisms using the same tissue or other tissues in other populations would be useful. We performed the assays in individual breeds and did not attempt to compare the breeds due to possible breed effect as we previously reported [23]. We elected to analyze *PRNP* expression in these breeds because of their economic and socio-cultural importance in Japan. Our sampling places (Kagoshima and Kumamoto prefectures) were preferred because they are the number one producers of JB and JBr breeds respectively. However, there are only a few BSE cases in JB breed (3 animals including 1 in Kumamoto) but the disease has not been diagnosed in JBr breed.

## Materials and Methods

### Animals, RNA isolation, cDNA synthesis and DNA isolation

We used the medulla oblongata tissue sampled from healthy 120 JB and 98 JBr from public slaughterhouses in Kagoshima and Kumamoto, Japan. Samples were taken randomly, but later coded according to age and sex. JB cattle aged between 25–36 months in (average 27–30 months) while JBr cattle aged between 22–159 months in (average 24–27 months). Total RNA was extraction from 500 mg of the dry freezed medulla oblongata tissue samples using either TRIZOL® Reagent (invitrogen™) or TriPure isolation reagents (Roche Diagnostics GmbH, Mannheim, Germany) following the manufacturers' instructions. Cleanup of the isolated RNA was done using the RNeasy® Mini Kit with RNase-Free DNase Set (QIAGEN, Tokyo, Japan) according to the manufacturer's instruction. Reverse transcription into cDNA was performed using 12.5-units of AMV Reverse Transcriptase (Promega K.K., Tokyo, Japan), 2.5-µl of random hexamer primer (TAKARA BIO INC., Otsu, Shiga, Japan) and 0.5 µg of the isolated RNA in a 50-µl reaction by a GeneAmp® PCR System 9700 (Applied Biosystems, Tokyo, Japan) at 30°C for 10 minutes, 37°C for 120 minutes and 99°C for 5 minutes. cDNA samples were stored at −20°C. Genomic DNA was isolated from 200 mg of the same tissue as that used for RNA isolation using PUREGENE™ Cell & Tissue kit (Gentra SYSTEMS, Minneapolis, MN, USA) according to the manufacturer's instructions. Concentration and purity of RNA, mRNA and the DNA were calculated by a GeneQuant 100 (GE Healthcare, UK) according to the manufacturer's instructions.

### *PRNP* genotyping and diplotype analysis

Genotyping with respect to the 23-bp indel within the promoter region and 12-bp indel within the first intron were amplified by polymerase chain reaction (PCR) using primer pairs GTGCCAGCCATGTAAGTG and TGGACAGGCACAATGGG (23-bp indel) also CTCGGTTTTACCCTCCTGGT and CACTTCCCAGCATGTAGCCACCA (12-bp indel). PCR was carried out in a final volume of 20 µl containing 0.5 units of *Ex Taq* (TAKARA), 40 ng of genomic DNA, 200 mmol/l each of the four deoxynucleoside triphosphates (dNTPs), 10 pmoles of each primer and 2.0 µl of 10× *Ex Taq* buffer (TAKARA). The mixture was first treated at 94°C for 2 min, followed by 28–35 cycles of denaturation at 94°C for 30 s, annealing temperature 56°C (23-bp) ; 62°C (12-bp) for 30 s, and extension at 72°C for 30 s and a final elongation step at 72°C for 5 min. All PCRs were carried out in the GeneAmp PCR System 9700 (Applied Biosystems). PCR products were separated on 10% polyacrylamide gels and visualized with ethidium bromide staining under ultra-violet (UV) light. From the genotypes, diplotypes for the two loci were constructed. Animals with the selected diplotypes were used in the gene expression analysis. The diplotypes were selected based on the objectives of our studies while considering the sample sizes. To avoid analysis of animals of the same origin (family relations, sex, age or farms), sampling of the medulla oblongata tissue was restricted to 10–15 samples per a sampling day. Information of these animals was known through a Japanese traceability system at http://www.nlbc.go.jp.

### Construction of a standard curve and real-time reverse-transcription PCR assay

Real-time PCR standard curves for each of the *PRNP* and an endogenous control *actin beta* gene (*ACTB*) were constructed by PCR products including the target sequence and its flanking sequence as a template. 10-fold dilution series of each standard was used to generate the real-time PCR standard curve. The PCR used to amplify this standard was carried out in the GeneAmp® PCR System 9700 using primer pairs listed in Table 4. Gene expression levels of the bovine *PRNP* and *ACTB* were quantitatively measured by the real-time PCR using primer pairs also listed in Table 4. Primer pairs were designed from the nucleotide sequences GenBank accession numbers AJ298878 (*PRNP*) and NM_173979 (*ACTB*) using Primer3 software [26]. The amplified bovine DNAs *PRNP* 153-bp and *ACTB* 169-bp were sequenced and confirmed to be identical with those from which primer pairs were designed. The real-time PCR was carried out in a 15-µl reaction volume containing 7.5-µl of SYBR® Green Real-time PCR Master Mix (TOYOBO, Osaka, Japan), 0.03-µM of each primer and 3.0-µl of each cDNA in the 7300 Real-Time PCR System (Applied Biosystems). Thermal cycling conditions were 95°C for 10 minutes followed by 40 cycles of 95°C for 10 seconds and 60°C for one minute. All reactions (standard, unknown samples and non-template control) were performed in duplicates on the same 96-well plate. Results reported here are averages of the duplicates. The *PRNP* expression level was normalized by dividing it with the *ACTB* expression level. *ACTB* was favored against *GAPDH* because the latter performed poorly in our medulla oblongata samples.

**Table 4:** List of primers used in this study.

Genes	sequence	Size	AT °C	Remarks
*PRNP*	F 5′-TCCCAGAGACACAAATCCAA-3′	153	60	External, construction of standard curve
	R 5′-ATCCTCCTCCAGGTTTTGGT-3′			
	F 5′-TCCAACTTGAGCTGAATCACA-3′	153	60	Internal, real-time PCR
	R 5′-CAGGTTTTGGTCGCTTCTTG-3′			
*ACTB*	F 5′-ACCATGTACCCCGGCATC-3′	169	60	External, construction of standard curve
	R 5′-TTGCTGATCCACATCTGCTG-3′			
	F 5′-ATCGAGGACAGGATGCAGAA-3′	154	60	Internal, real-time PCR
	R 5′-CACATCTGCTGGAATGTGGA-3′			

AT: Annealing temperature; *PRNP*: Prion protein gene; *ACTB*: Actin beta gene.

### Statistical analyses

Raw data were developed by spreadsheets and later subjected to the GLM procedure of SAS® Proprietary Software, Release 8.2 (SAS Institute Inc. Cary, NC, USA) for statistical analyses using a linear model including diplotypes, sex, age, region/farms and their interactions as shown below. We performed the independent t-tests to support the *PRNP* expression of the normally distributed data. All decisions were made following the differences in dependent variables but also considered the values of coefficient of determination (R^2^) and the t grouping. R^2^ values ranging from 0.6725–0.9085 and *P*-values<0.05 were considered significant in our analyses.
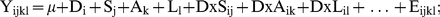
Where as :

Y_ijkl_ = Expression
μ = Overall mean
D_i_ = Random effect of the i^th^ diplotype
S_j_ = Random effect of the j^th^ sex
A_k_ = Random effect of the k^th^ age
L_l_ = Random effect of the l^th^ location
E_ijkl_ = Random residual effect of each observation (error effect)
